# Facile Preparation of Three-Dimensional Cubic MnSe_2_/CNTs and Their Application in Aqueous Copper Ion Batteries

**DOI:** 10.3390/nano14201621

**Published:** 2024-10-10

**Authors:** Junjun Wang, Linlin Tai, Wei Zhou, Han Chen, Jingxiong Liu, Shaohua Jiang

**Affiliations:** 1 College of Liling Ceramic, Hunan University of Technology, Zhuzhou 412007, China; 2 Hunan Key Laboratory of Applied Environmental Photocatalysis, Changsha University, Changsha 410022, China; zhouwei_csu@163.com (W.Z.); 18900753550@163.com (H.C.); 3 Jiangsu Co-Innovation Center of Efficient Processing and Utilization of Forest Resources, International Innovation Center for Forest Chemicals and Materials, College of Materials Science and Engineering, Nanjing Forestry University, Nanjing 210037, China

**Keywords:** aqueous battery, MnSe_2_, carbon nanotubes, Cu-ion battery

## Abstract

Transition metal sulfide compounds with high theoretical specific capacity and excellent electronic conductivity that can be used as cathode materials for secondary batteries attract great research interest in the field of electrochemical energy storage. Among these materials, MnSe_2_ garners significant interest from researchers due to its unique three-dimensional cubic structure and inherent stability. However, according to the relevant literature, the performance and cycle life of MnSe_2_ are not yet satisfactory. To address this issue, we synthesize MnSe_2_/CNTs composites via a straightforward hydrothermal method. MnSO_4_·H_2_O, Se, and N_2_H_4_·H_2_O are used as reactants, and CNTs are incorporated during the stirring process. The experimental outcomes indicate that the fabricated electrode demonstrates an initial discharge specific capacity that reaches 621 mAh g^−1^ at a current density of 0.1 A g^−1^. Moreover, it exhibits excellent rate capability, delivering a discharge specific capacity of 476 mAh g^−1^ at 10 A g^−1^. The electrode is able to maintain a high discharge specific capacity of 545 mAh g^−1^ after cycling for 1000 times at a current density of 2 A g^−1^. The exceptional electrochemical performance of the MnSe_2_/CNTs composites can be ascribed to their three-dimensional cubic architecture and the 3D CNT network. This research aids in the progression of aqueous Cu-ion cathode materials with significant potential, offering a viable route for their advancement.

## 1. Introduction

Propelled by carbon-neutrality policies, the widespread adoption of renewable energy has become an inevitable direction for development. However, new energy sources, such as wind, solar, tidal, and geothermal energy, are limited by natural conditions such as climate, latitudinal location, topography, etc., and there is a serious imbalance between supply and demand in time and space. Therefore, the vigorous development of electrochemical energy storage systems is currently recognized as the most effective solution strategy, such as Zn-ion batteries [1], supercapacitor [2,3,4], Li-ion battery [5], ammonium-ion batteries [6,7,8], hybrid battery [9], microbial fuel cell [10], Li-S batteries [11], zinc-ion hybrid supercapacitors [12], and so on. Lithium-ion batteries are predominant across various types of energy storage technologies, particularly in applications involving new energy vehicles, electronic devices, and renewable energy storage systems. This dominance is attributed to their exceptional high energy density, robust power output capabilities, and well-established industrial production processes. These advantages have enabled lithium-ion batteries to exhibit significant competitiveness and reliability across a wide range of applications [13,14,15]. Despite the extensive application of lithium-ion batteries across various fields, their high costs and less-than-optimal safety performance have constrained the further development of this technology. In contrast, among the many battery technologies that have emerged in recent years, aqueous ion batteries have rapidly gained research prominence and widespread attention. This is as a result of their cost-effectiveness, exceptional stability, superior safety features, and sustainability advantages [16,17]. Currently, a variety of metal ions, for example, Zn^2^⁺, Na⁺, K⁺, Mg^2^⁺, Cu^2^⁺, and Al^3^⁺, are being explored as charge carriers in conventional aqueous batteries [18,19]. Among these metals, copper (Cu) stands out due to its high reaction potential, impressive theoretical specific capacity (844 mA h/g and 7558 mA h/cm^3^), and significant natural abundance [20,21].

Wu et al. [22] reported an innovative aqueous secondary battery utilizing Cu^2+^ as the charge carrier, demonstrating excellent redox activity for energy storage. Their findings revealed a sequential S-CuS-Cu_2_S conversion mechanism in the sulfur electrode, facilitating a four-electron reaction process. The fabricated copper–sulfur cell exhibited a remarkable electrochemical performance, achieving a specific capacity of up to 3044 mAh g^−1^ at a current density of 100 mA g^−1^ and retaining 72% of its initial capacity after 1200 cycles at an elevated current density of 12.5 A g^−1^. In addition, Zhang J. S. et al. [23] conducted a systematic investigation into the effects of anion exchange on the kinetic properties of CuS_1−x_Se_x_. By combining electrochemical analysis with structural and spectral studies, they highlighted that the anion exchange between S and Se in CuS_1−x_Se_x_ not only mitigated phase transitions but also enhanced electron and ion transport by reducing energy barriers. This improvement facilitated stable copper ion storage and significantly boosted electrochemical performance. Specifically, self-supported CuS_0.5_Se_0.5_ nanosheets delivered a specific capacity of up to 491 mAh g^−1^ and maintained 80% of their reversible capacity at a high current density of 20 A g^−1^, outperforming CuS. Despite these advancements, the development of aqueous copper batteries remains in its early stages. Among these, the application of transition metal sulfide cathode materials in aqueous copper batteries is still in its initial stages of development. The key to their further development lies in the advancement of high-performance electrode materials and a deeper understanding of the mechanisms underlying copper ion energy storage.

In the contemporary research landscape, transition metal sulfide compounds have surfaced as potential options for energy storage applications because of their distinct chemical characteristics, including extensive redox chemistry, which enables a wide array of practical uses [24,25]. Among the various transition metal compounds, selenides and sulfides particularly excel due to their exceptional electrocatalytic activity, high electrical conductivity, and robust metal bonding characteristics [26,27]. Recently, extensive studies have been conducted on metal selenides and sulfides [28,29,30,31,32,33,34,35], demonstrating their substantial stability and performance in energy storage systems. Selenium (Se) exhibits lower ionization energy and better electrical conductivity compared to sulfur (S), making the substitution of Se for oxygen (O) atoms in manganese compounds a focal point for researchers [36]. Among these selenides, manganese diselenide distinguishes itself due to its low cost, low toxicity, and excellent catalytic properties. As a result, it is extensively used in electrode materials and oxygen reduction catalysts for lithium- and sodium-ion batteries. However, the application of manganese diselenide in aqueous copper-ion batteries remains unexplored. Therefore, the utilization of manganese diselenide as a cathode material for aqueous copper-ion batteries warrants investigation. Regrettably, manganese diselenide faces challenges such as sluggish kinetics and volume expansion [37]. To tackle these challenges, carbon-based materials, particularly one-dimensional carbon nanotubes (CNTs), are extensively utilized as conductive additives [38]. Owing to their distinctive cross-linking capabilities and disordered structural arrangement, carbon nanotubes enhance the mechanical stability of composite materials while simultaneously facilitating a broader distribution of active sites [39,40]. Consequently, incorporating carbon nanotubes into manganese-based compounds can greatly enhance the stability and electrical conductivity of these materials.

To improve the electrochemical performance of MnSe_2_ cathode materials in aqueous copper ion batteries, we successfully synthesized MnSe_2_/CNTs (MSCN) composites via a hydrothermal method. Se, MnSO_4_, and CNTs served as the sources of selenium, manganese, and carbon, respectively. The CNTs were uniformly adhered to the surface of the MnSe_2_ cubes, effectively mitigating electrode volume expansion and improving copper ion conductivity. The MSCN composite shows an impressive starting discharge specific capacity of 621 mAh g^−1^ under a current density of 0.1 A g^−1^. It also reveals a significant rate performance, sustaining a discharge specific capacity of 477 mAh g^−1^ when exposed to a current density of 10 A g^−1^. Additionally, after 1000 cycles at a current density of 2 A g^−1^, the composite maintains a high discharge specific capacity of 545 mAh g^−1^. The MSCN cathode presents a promising avenue for rapid and durable copper ion storage.

## 2. Materials and Methods

### 2.1. Materials

Selenium powder (99.99%) and manganese sulfate monohydrate (99%) were sourced from Sinopharm Chemical Reagent Co. (Shanghai, China). Citric acid monohydrate (99.5%) and carbon nanotubes (≥95%) were acquired from Shanghai Aladdin Biochemical Technology Co. (Shanghai, China). Hydrazine hydrate (80%) was obtained from Hunan Huihong Reagent Co. Anhydrous ethanol was provided by Tianjin Fuyu Fine Chemical Industry (Tianjin, China), while acetylene black, polyvinylidene fluoride, and N-methyl pyrrolidone were procured from Advanced Technology Industry Corporation(Changsha, China). All solutions were prepared using distilled water as the solvent, and all raw materials employed were of analytical grade, used without further purification.

### 2.2. Synthesis of MnSe_2_/CNTs Composites

Firstly, to synthesize the desired material, 0.338 g of manganese sulfate monohydrate, 0.3158 g of selenium powder, and 3 g of citric acid were dissolved in 44 mL of distilled water and stirred for 30 min. Following this, 16 mL of hydrazine hydrate were introduced to the mixture, followed by an additional hour of stirring to ensure thorough mixing. Concurrently, 50 mg of CNTs were introduced into the mixed solution and subjected to ultrasonication for 30 min, resulting in a homogeneous mixture. The solution was then placed into a 100 mL Teflon-lined autoclave and heated at 180 °C for a duration of 12 h. Once the autoclave had cooled to ambient temperature, the resulting black precipitate was collected and meticulously washed with distilled water and anhydrous ethanol. The cleaned precipitate was subsequently dried in a vacuum at 60 °C for 12 h. The final samples were labeled as MSCN. To facilitate comparison, the CNT quantity was varied to 20 mg and 80 mg, while keeping other conditions constant. These three samples were accordingly named MSCN-1, MSCN-2, and MSCN-3. In the absence of carbon nanotubes (CNTs), the synthesized sample consisted solely of MnSe_2_.

### 2.3. Materials Characterizations

The composition and crystal structure of the synthesized samples were analyzed using X-ray diffraction (XRD) with a Rigaku ULTIMA IV X-ray powder diffractometer (Tokyo, Japan). The X-ray generator operated at 3 kW, with a Cu Kα radiation source (λ = 0.154056 nm). Continuous scanning was performed at a speed of 5°/min across a range from 10° to 90°.

The composition and chemical state of the synthesized samples were further examined by X-ray photoelectron spectroscopy (XPS), using a Thermo Fisher Scientific K-Alpha spectrometer (Waltham, MA, USA). The system employed an Al Kα source, with an excitation energy of 1486.8 eV, a test spot area adjustable between 50 and 400 µm (in 5 µm steps), a tube voltage of 15 kV, and a tube current of 10 mA. The data-acquisition step size was 0.05 eV, and the resulting spectra were analyzed using XPS PEAK 41 software. To investigate the morphology and microstructure of the samples and electrodes, scanning electron microscopy (SEM) and transmission electron microscopy (TEM) were utilized. SEM analysis was conducted using a Carl Zeiss Sigama 300 field emission SEM equipped with a backscattered electron probe (BSE) and an X-ray energy-dispersive spectrometer (EDS). TEM analysis was performed using a Tecnai G2 F20 field-emission high-resolution transmission electron microscope from FEI (Hillsboro, OR, USA).

### 2.4. Electrochemical Measurements

MSCN-1, MSCN-2, MSCN-3, and copper foil were utilized as working and counter electrodes, respectively. A cellulose membrane served as the separator, and the electrolyte was composed of 0.5 mol L^−1^ CuSO_4_ solution. These components were assembled into a CR-2025 coin cell for subsequent electrochemical performance testing. The MSCN-x (x = 1, 2, 3), acetylene black, and polytetrafluoroethylene binder (PVDF) were homogeneously mixed in an 8:1:1 mass ratio, respectively. Subsequently, to facilitate the milling process, N-methyl pyrrolidone (NMP) was introduced as a solvent. Stainless steel was chosen as the current collector, upon which the aforementioned mixed slurry was uniformly applied. The coated substrate was then placed in a vacuum-drying oven, where it was maintained at 60 °C for a duration of 24 h to ensure thorough drying. Finally, the dried stainless-steel foil was punched into circular electrodes with a diameter of 12 mm, using a slicing machine, and the prepared electrodes and copper foil anodes were assembled into CR-2025 coin cells. Electrochemical tests, normalized to the mass of the active cathode material, were conducted using a CHI660E electrochemical workstation (Austin, TX, USA) and a Neware CT-4008T battery tester (Shenzhen, China).

## 3. Results and Discussion

**Figure 1:**
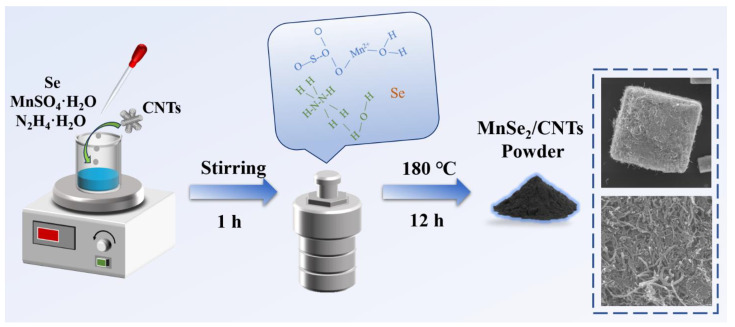
Diagrammatic representation of the fabrication process for MSCN-x (x = 1, 2, 3) composite structures.

A straightforward hydrothermal treatment approach was employed, utilizing a temperature of 180 °C for 12 h. In this process, MnSO_4_·H_2_O and Se served as the sources of manganese and selenium, respectively, with the addition of carbon nanotubes during the stirring procedure. The uniform distribution of components in the solution facilitates the improved synthesis of MnSe_2_/CNTs composites. The detailed synthesis procedure is depicted in Figure 1.

The potential reactions occurring during the hydrothermal synthesis process are elucidated as follows:

MnSO_4_·H_2_O + 2N_2_H_4_·H_2_O + 2Se → MnSe_2_ + (NH_4_)_2_SO_4_ + 3H_2_O + N_2_↑ (1)

**Figure 2:**
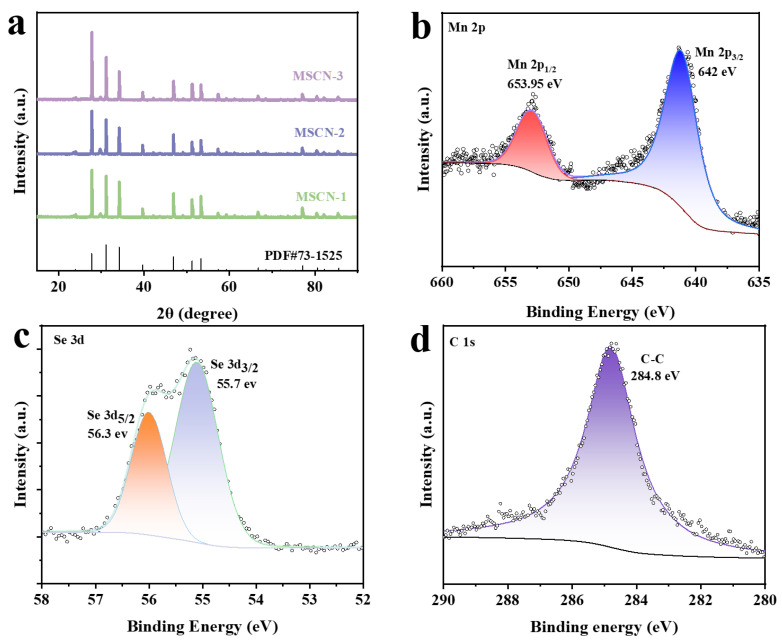
(**a**) XRD patterns of MSCN-1, MSCN-2, and MSCN-3. (**b**–**d**) High-resolution spectra of Mn 2p, Se 3d, and C 1s of the MSCN-2.

In the hydrothermal reaction with Mn^2^⁺, selenium powder was reduced by hydrazine hydrate, while citric acid primarily functioned to neutralize the excess alkaline hydrazine hydrate. MSCN-x (x = 1, 2, 3) was characterized and analyzed using X-ray diffraction (XRD) and X-ray photoelectron spectroscopy (XPS). As illustrated in Figure 2a, the X-ray diffraction (XRD) patterns of the MSCN-x (x = 1, 2, 3) composite materials align precisely with the reference data (PDF#73-1525) [41]. The diffraction peaks observed at 24.27°, 27.78°, 31.14°, 34.19°, 39.69°, 42.21°, 46.92°, 49.14°, and 51.29° correspond to the (111), (200), (210), (211), (220), (221), (311), (222), and (023) crystallographic planes, respectively, indicating high crystallinity. The above analysis indicates that no significant impurity phases were detected, and the incorporation of carbon nanotubes did not interfere with the synthesis of MnSe_2_. In addition to the crystalline surface corresponding to MnSe_2_, a low-intensity amorphous bump around 24.5° is clearly observed, attributed to the (002) plane of amorphous carbon. No other noticeable impurity phases were detected in the pattern.

To gain a deeper understanding of the chemical-bonding states and elemental composition of the synthesized materials, the samples were analyzed by X-ray photoelectron spectroscopy (XPS), which is often used to measure the elemental composition, as well as the chemical and electronic state of the atoms of materials [42,43,44,45]. As shown in Figure 2b–d, the XPS results confirm the presence of Mn 2p and Se 3d elements, with carbon (C 1s) adsorbed on the surface. As depicted in Figure 2c, two prominent peaks were observed at 642 eV and 653.95 eV, corresponding to Mn 2p_3/2_ and Mn 2p_1/2_, respectively [46]. Figure 2d displays the Se elemental analysis, showing peaks at 55.7 eV (Se 3d5/2) and 56.3 eV (Se 3d3/2), which indicate a chemical bond between Mn and Se [47]. As presented in Figure 2d, the high-resolution C 1s spectrum detected at 284.8 eV signifies the presence of C-C bonds [48].

**Figure 3:**
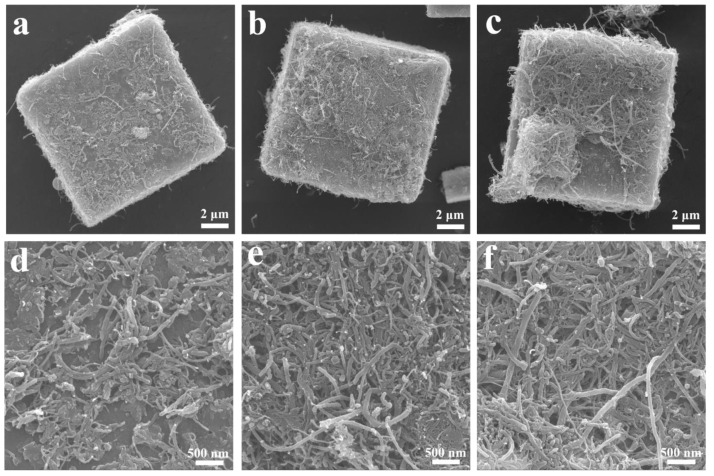
SEM images of (**a**,**d**) MSCN-1, (**b**,**e**) MSCN-2, and (**c**,**f**) MSCN-3.

The morphology of MSCN-x (x = 1, 2, 3) was investigated with scanning electron microscopy (SEM), which is effective for investigating the surfaces of materials [49,50,51]. As illustrated in Figure 3a–f, the samples exhibit a distinctive micro-sized cubic morphology, with varying amounts of carbon nanotubes (CNTs) attached to their surfaces due to differences in CNT concentration. Figure 3c reveals that MSCN-3 exhibits agglomerated CNTs on its surface. In contrast, MSCN-1 displays a heterogeneous distribution with sparse CNTs, as illustrated in Figure 3a. Meanwhile, MSCN-2 demonstrates embedded homogeneous CNTs, as evidenced in Figure 3b. Upon closer examination, as illustrated in Figure 3f, significant CNT aggregation is evident in MSCN-3, which is likely contributing to the observed effects on electrochemical performance, as discussed in subsequent sections. In contrast, Figure 3d shows that the CNTs in MSCN-1 are not uniformly distributed on the cubes. Conversely, the surface of MSCN-2 in Figure 3e appears more homogeneous, with fewer CNTs aggregations, and this is a critical factor for its superior electrochemical performance. For a clearer and more intuitive comparison, Supplementary Figure S1 reveals that, without the addition of CNTs, the structure remains a clean cubic form. The inclusion of surface CNTs did not alter the intrinsic 3D MnSe_2_ structure. The consistent homogeneity of CNTs in MSCN-2 is corroborated by multiple observations in these figures.

**Figure 4:**
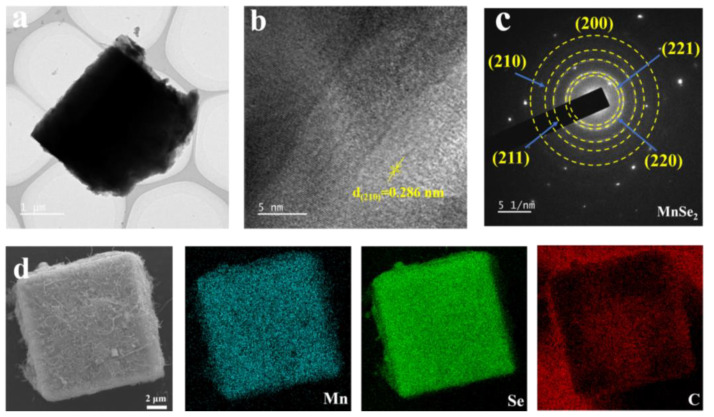
(**a**) HAADF-STEM images of the MSCN-2, (**b**) HR-TEM image, (**c**) SAED pattern, and (**d**) corresponding EDS elemental mapping of MSCN-2.

These homogeneous CNTs provide more edges and faces, effectively increasing the overall surface area and thereby enhancing electrochemical performance. Transmission electron microscopy (TEM) analysis of MSCN-2, as depicted in Figure 4a–c, shows a distinct lattice spacing of 2.86 Å, which matches the (210) plane of MnSe_2_, as confirmed by high-resolution TEM (Figure 4b). The patterns from selected-area electron diffraction (SAED) clearly show distinct diffraction rings, as depicted in Figure 4c, corresponding to the (221), (220), (211), and (210) planes of MnSe_2_, respectively, which align perfectly with the XRD results, thus confirming the excellent crystallization of MnSe_2_. Additionally, the elemental mapping of the MSCN-2 composites using energy-dispersive spectroscopy (EDS), as illustrated in Figure 4d, indicates a uniform distribution of Mn, Se, and C elements within MSCN-2. In addition, the energy-dispersive X-ray spectroscopy (EDS) image (Supplementary Figure S7) shows that the atomic ratio of Mn to Se is approximately 2:1, which is consistent with the results from elemental mapping. This homogeneous distribution significantly increases conductive pathways, mitigates volume expansion, facilitates electrolyte diffusion, and improves electron transfer, thereby enhancing the electrochemical storage capacity of the material.

**Figure 5:**
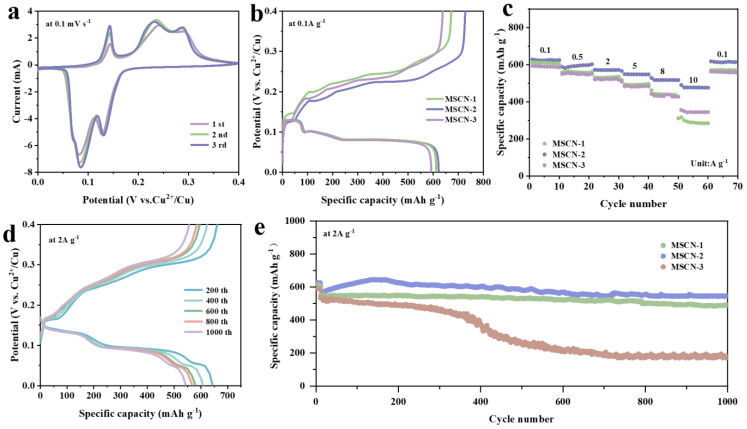
(**a**) The cyclic voltammetry (CV) curves of MSCN-2 during the initial three cycles. (**b**) Initial GCD curves at 0.1 A g^−1^ of the MSCN-x (x = 1, 2, 3). (**c**) Rate capability. (**d**) MSCN-2 GCD curves of different cycles. (**e**) Long-term cycling performance at 2 A g^−1^.

Cyclic voltammetry (CV) measurements of the MSCN-2 electrodes were conducted at a scan rate of 0.1 mV s^−1^, as illustrated in Figure 5a. The cyclic voltammograms (CV) of the MSCN-1 (Supplementary Figure S2) and MSCN-3 (Supplementary Figure S3) electrodes exhibit similar characteristics to those observed in the MSCN-2 electrode. All CV curves displayed distinct oxidation–reduction peaks and exhibited similar profiles. Specifically, two pairs of pronounced anodic and cathodic peaks were observed near 0.14/0.25 V and 0.08/0.13 V, individually, which results from the reversible insertion and extraction of Cu^2+^ ions at the MSCN anode [52]. Moreover, the MSCN-2 electrode demonstrated higher peak currents at redox potentials compared to MSCN-1 and MSCN-3 electrodes, suggesting superior electrochemical activity, and such enhanced activity facilitates the kinetics of Cu^2+^ (de)insertion, thereby resulting in the highest specific capacity among the three electrodes. In Figure 5b, the initial charge and discharge profiles for MSCN-1, MSCN-2, and MSCN-3 at a current density of 0.1 A g^−1^ are depicted. A noticeable voltage plateau is evident in the 0~0.4 V range, which corresponds closely with the previously discussed CV results. Notably, the MSCN-2 electrode achieved an initial discharge specific capacity of 621 mAh g^−1^, exceeding the specific capacities of MSCN-1 (612 mAh g^−1^) and MSCN-3 (592 mAh g^−1^). Figure S4 presents the charge/discharge curves of MnSe_2_ for the initial three cycles at a current density of 0.1 A g⁻^1^. The specific discharge capacities recorded were 585 mAh g⁻^1^ for the first cycle, 396 mAh g⁻^1^ for the second cycle, and 424 mAh g⁻^1^ for the third cycle. These values are lower than the specific discharge capacities of MSCN-1, MSCN-2, and MSCN-3. This evidence further underscores the significant role that CNT incorporation plays in enhancing the electrochemical performance of the material.

The rate performance of MSCN-1, MSCN-2, and MSCN-3 electrodes is depicted in Figure 5c. Among the three, the MSCN-2 electrode demonstrates a superior rate performance, maintaining an excellent discharge capacity across various current densities. The specific discharge capacities at current densities of 0.1, 0.5, 2, 5, 8, and 10 A g^−1^ were 622, 593, 570, 547, 517, and 476 mAh g^−1^. While the multiplier performance of MSCN-1 and MSCN-3 was not satisfactory, the discharge specific capacity of MSCN-1 at the same multiplier current was 611, 559, 530, 491, 440, and 288 mAh g^−1^, and that of MSCN-1 at the same multiplier current was 590, 549, 523, 481, 430, and 343 mAh g^−1^. respectively. Notably, under elevated current densities (at 10 A g^−1^), the discharge specific capacity of the MSCN-2 electrode significantly exceeded that of MSCN-1 (343 mAh g^−1^) and MSCN-3 (286 mAh g^−1^). Furthermore, upon reverting the current density back to 0.1 A g^−1^, the MSCN-2 electrode retained a discharge specific capacity of 615 mAh g^−1^, while the discharge specific capacities of MSCN-1 and MSCN-3 under the same conditions were 571 and 559 mAh g^−1^. These results demonstrate the excellent rate stability of the MSCN-2 electrode. Figure 5d illustrates the cycling performance of the MSCN-2 electrode when subjected to a current density of 2 A g^−1^, showing reversible discharge specific capacities of 644, 608, 580, 569, and 544 mAh g^−1^ after 200, 400, 600, 800, and 1000 cycles, respectively. This indicates the excellent cycling stability of the MSCN-2 electrode. In comparison to the MSCN-1 and MSCN-3 electrodes, MSCN-2 exhibits superior long-term cycling performance when operated at a current density of 2 A g^−1^, as illustrated in Figure 5e. Initially, the MSCN-x (x = 1, 2, 3) electrodes exhibit similar specific capacities, likely due to the gradual electrochemical activation process occurring within the MSCN electrodes [52]. Remarkably, following 200 cycles, the MSCN-2 electrode exhibits a substantially higher specific capacity compared to both MSCN-1 and MSCN-3. After 400 cycles, a sharp decline in specific capacity is observed for the MSCN-3 electrode. The post-cycled X-ray spectroscopy (EDS) image reveals a significant decrease in the elemental content of Mn and Se compared to pre-cycling levels, with Se and Mn percentages at 2.9% and 0.3%, respectively. This reduction may be the primary reason for the sudden decline in the capacity of the MSCN-3 electrode after 400 cycles. In contrast, the capacities of the MSCN-1 and MSCN-2 electrodes remain more stable. By the 1000th cycle, the specific capacity of the MSCN-3 electrode drops to 179 mAh g^−1^, while the MSCN-1 and MSCN-2 electrodes retain specific capacities of 491 and 545 mAh g^−1^, respectively. The moderate content of carbon nanotubes (CNTs) in the MSCN-2 electrode provides a distinct advantage, enabling it to achieve a high reversible specific capacity.

**Figure 6:**
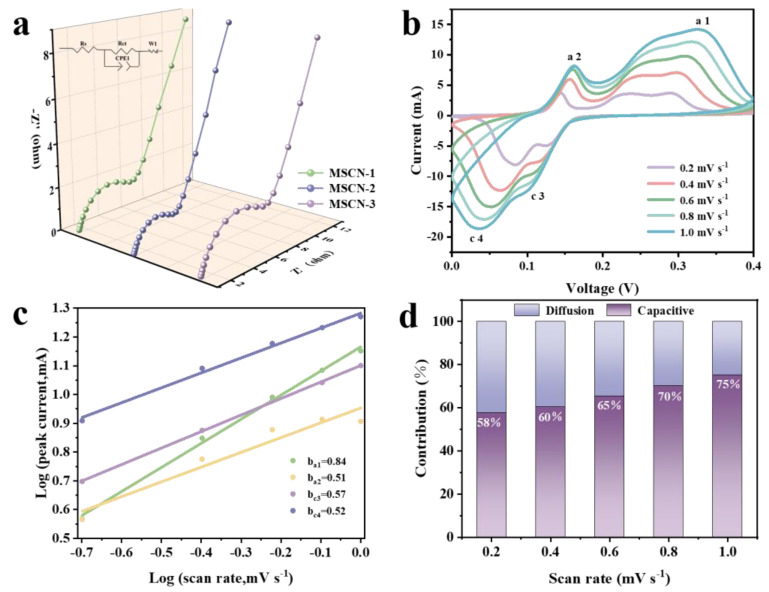
(**a**) Electrochemical impedance spectra (EIS) of MSCN-x (x = 1, 2, 3) electrodes. (**b**) CV curves of MSCN-2 at different scanning speeds. (**c**) Plots of log (i) vs. log (v) for Peak1~Peak 4 of MSCN-2. (**d**) Capacitive contribution ratios at different scan rates of MSCN-2.

The electrochemical performance of the MSCN-1, MSCN-2, and MSCN-3 electrodes was thoroughly evaluated, as illustrated in Figure 6. Electrochemical impedance spectroscopy (EIS) tests were performed on MSCN-x (x = 1, 2, 3) electrodes, with the resulting Nyquist plots displayed in Figure 6a. The impedance curve consists of two distinct components: a semicircle observed at mid- and high-frequencies and a diagonal line at low frequencies. The high-frequency semicircle corresponds to the charge-transfer resistance, while the low-frequency diagonal line reflects the Warburg impedance. Notably, Among the electrodes, the MSCN-2 electrode demonstrates the lowest charge-transfer resistance (Rct) value of 1.15 Ω, in comparison to MSCN-1, with 1.53 Ω, and MSCN-3, with 6.37 Ω, indicating a more efficient charge-transfer process in the MSCN-2 electrode. Figure 6b presents the cyclic voltammetry (CV) curves of the MSCN-2 cathode material at various scan rates. Notably, two distinct sets of redox peaks can be observed, corresponding to the Cu^2+^ insertion/extraction processes. Additionally, as the scan rate increases, the polarization phenomenon causes the oxidation peaks to gradually shift to the right and the reduction peaks to the left, while the overall shape of the CV curves remains unchanged. The charge diffusion kinetics were analyzed using the equation I = av^b^, where I represents the current, v is the scan rate, and a and b are fitting parameters. When the b value is close to 0.5, the reaction process is predominantly diffusion-controlled; when the b value approaches 1.0, it is controlled by pseudocapacitive behavior [53,54]. As shown in Figure 6c, the electrochemical reaction appears to be influenced by both pseudocapacitive behavior and diffusion, as indicated by the values close to 0.5. The pseudocapacitive and diffusion-controlled capacities can be calculated using the equation i = k1v + k2v1/2, where k1v and k2v1/2 represent the pseudocapacitive and diffusion contributions, respectively. Figure 6d illustrates the capacitance contribution of the MSCN-2 cathode material at different scan rates, increasing from 58% to 75% as the scan rate increases from 0.2 mV s^−1^ to 1.0 mV s^−1^. Furthermore, according to the CV capacitance contribution curve at 0.2 mV s^−1^, the capacitance contribution at this scan rate is determined to be 42% (Supplementary Figure S5).

**Figure 7:**
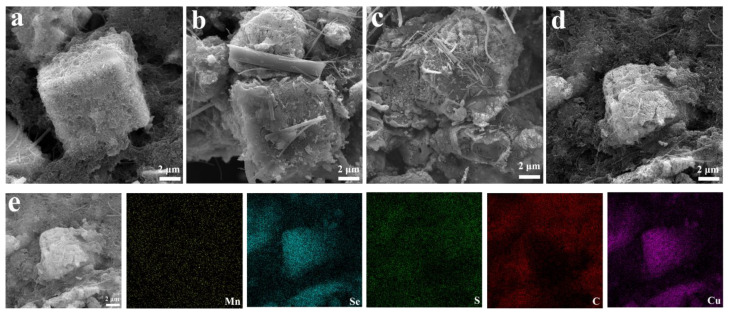
Scanning electron micrographs of the MSCN-2 electrode after varying numbers of charge–discharge cycles: (**a**) pristine, (**b**) 300 cycles, (**c**) 600 cycles, and (**d**) 900 cycles. (**e**) Energy-dispersive X-ray spectroscopy (EDS) mapping illustrating the elemental distribution on the electrode surface after 900 charge–discharge cycles.

To investigate the morphological evolution after different cycling intervals, the morphology of the MSCN-2 electrode in its pristine state, as well as after 300, 600, and 900 cycles, was analyzed using scanning electron microscopy (SEM), as illustrated in Figure 7a–d. In the pristine state, the MSCN cubes were uniformly distributed on the collector electrode, with carbon nanotubes (CNTs) evenly wrapping the cubes’ surfaces without any agglomeration, and there are no other impurities in the cube to indicate that the structure behaves more completely. The morphology of the MSCN-2 electrode after 300 cycles remained nearly unchanged compared to the original pristine sheet, However, despite the presence of some impurities and glass fibers around the cubes, the overall cubic structure remains unaffected, indicating robust structural stability during cycling. However, after 600 cycles, the MSCN-2 electrode exhibited slight chalking and agglomeration. The disruption of the cubic structure was the main reason for the degradation of its electrochemical properties. As charging and discharging continued, the pulverization of the morphology became more pronounced after 900 cycles, and the cubic structure had completely collapsed and was surrounded by impurities. The elemental distribution, depicted in Figure 7e, indicates the presence of Cu, in addition to Mn, Se, S, and C, within the electrode material, suggesting successful incorporation of Cu^2^⁺ ions into the MSCN-2 structure. According to the EDS total spectrum (Supplementary Figure S6), it can be clearly seen that the content of C is the most, accounting for 90.4%, and the remaining Cu and Mn account for 5.8% and 0.3%, respectively. It shows that the content of Cu^2+^ is increasing with the insertion and detachment of Cu^2+^ as the cycle proceeds, while the content of Mn is decreasing. During the cycling of the MSCN-2 electrodes, no cracking of the layers was observed. Considering the beneficial properties of Cu^2^⁺ storage and the protective role of CNTs in maintaining the cubic structure, it can be inferred that the use of MSCN-2 composites as electrodes enhances the durability of copper-ion batteries.

## 4. Conclusions

In summary, three-dimensional cubic MnSe_2_/CNTs composites were produced using a simple hydrothermal technique and efficiently utilized in aqueous copper-ion batteries. The synthesized composites exhibit a distinctive architecture, wherein the three-dimensional cubic MnSe_2_ structures are uniformly encased by CNTs. The presence of CNTs does not alter the morphology and structure of MnSe_2_; instead, their incorporation significantly enhances the electrical conductivity and mitigates volume changes, thereby ensuring the structural integrity of the composites. Among the MnSe_2_/CNTs (MSCN) composites synthesized, the MSCN-2 composite demonstrated the lowest charge-transfer resistance (Rct), i.e., only 1.15 Ω. The initial discharge specific capacity was found to be 621 mAh g^−1^ at a current density of 0.1 A g^−1^, and it maintained a significant specific capacity of 476 mAh g^−1^ even at 10 A g^−1^. Furthermore, after 1000 cycles, the MSCN-2 composite maintained a specific capacity of 545 mAh g^−1^ at a current density of 2 A g^−1^. This study underscores the potential of MnSe_2_/CNTs electrodes as a viable strategy for the development of high-performance aqueous copper-ion battery cathode materials.

## Data Availability

The original contributions presented in the study are included in the article/Supplementary Material, further inquiries can be directed to the corresponding authors.

## Supplementary Materials

The following supporting information can be downloaded at https://www.mdpi.com/article/10.3390/nano14201621/s1, Figure S1: SEM images of MnSe_2_; Figure S2: The cyclic voltammetry (CV) curves of MSCN-1 during the initial three cycles; Figure S3: The cyclic voltammetry (CV) curves of MSCN-3 during the initial three cycles; Figure S4: GCD curves at 0.1 A g^−1^ of the MnSe_2_; Figure S5: Capacitive contribution at 0.2 mV s^−1^; Figure S6: EDS Sum Spectrum of MSCN-3; Figure S7: EDS Sum Spectrum of MSCN-2.
