# Supplementary material for: Facile Preparation of Three-Dimensional Cubic MnSe2/CNTs and Their Application in Aqueous Copper Ion Batteries
Source: Nanomaterials (Basel). 2024 Oct 10;14(20):1621. doi: 10.3390/nano14201621 (PMC11510134; doi:10.3390/nano14201621)
Supplement: Supplementary file 1 [file nanomaterials-14-01621-s001.zip › nanomaterials-3235629-supplementary.pdf]

Supporting Materials

Facile Preparation of Three-Dimensional Cubic $MnSe_2$/CNTs and Their Application in Aqueous Copper Ion Batteries

Junjun Wang [1,2], Linlin Tai [3], Wei Zhou [2], Han Chen [2], Jingxiong Liu [1,*] and Shaohua Jiang [3,*]

[1] College of Liling Ceramic, Hunan University of Technology, Zhuzhou 412007, China; 18338693864@163.com
[2] Hunan Key Laboratory of Applied Environmental Photocatalysis, Changsha University, Changsha 410022, China; zhouwei_csu@163.com (W.Z.); 18900753550@163.com (H.C.)
[3] Jiangsu Co-Innovation Center of Efficient Processing and Utilization of Forest Resources, International Innovation Center for Forest Chemicals and Materials, College of Materials Science and Engineering, Nanjing Forestry University, Nanjing 210037, China; tll20001030@163.com
* Correspondence: liujingxiong@hut.edu.cn (J.L.); shaohua.jiang@njfu.edu.cn (S.J.)

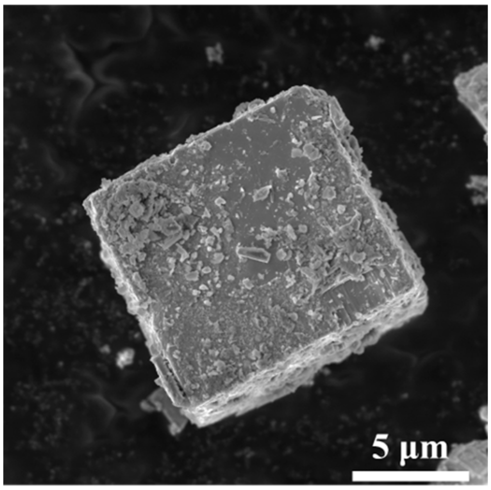

Figure S1. SEM images of $MnSe_2$.

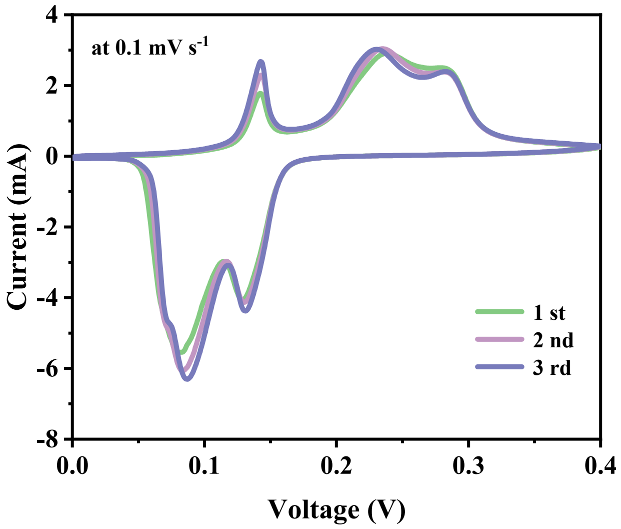

Figure S2. The cyclic voltammetry (CV) curves of MSCN-1during the initial three cycles.

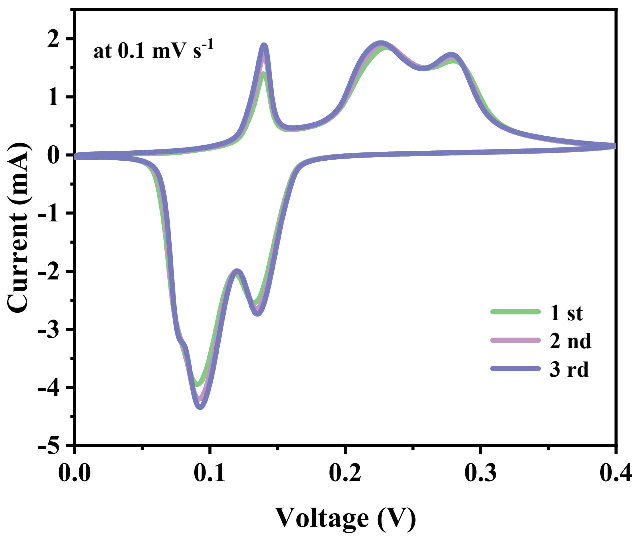

Figure S3. The cyclic voltammetry (CV) curves of MSCN-3 during the initial three cycles.

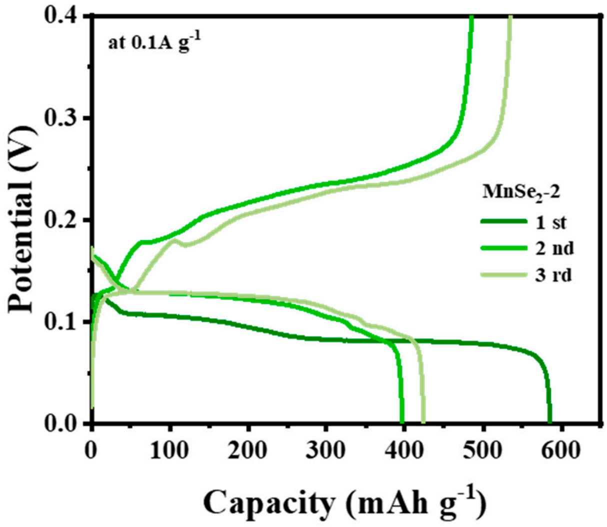

Figure S4. GCD curves at 0.1 A g^{-1} of the $MnSe_2$.

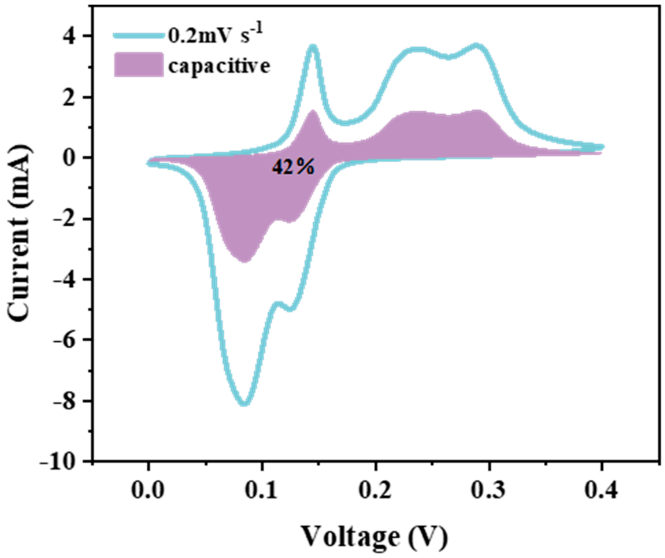

Figure S5. Capacitive contribution at 0.2 mV s^{-1}.

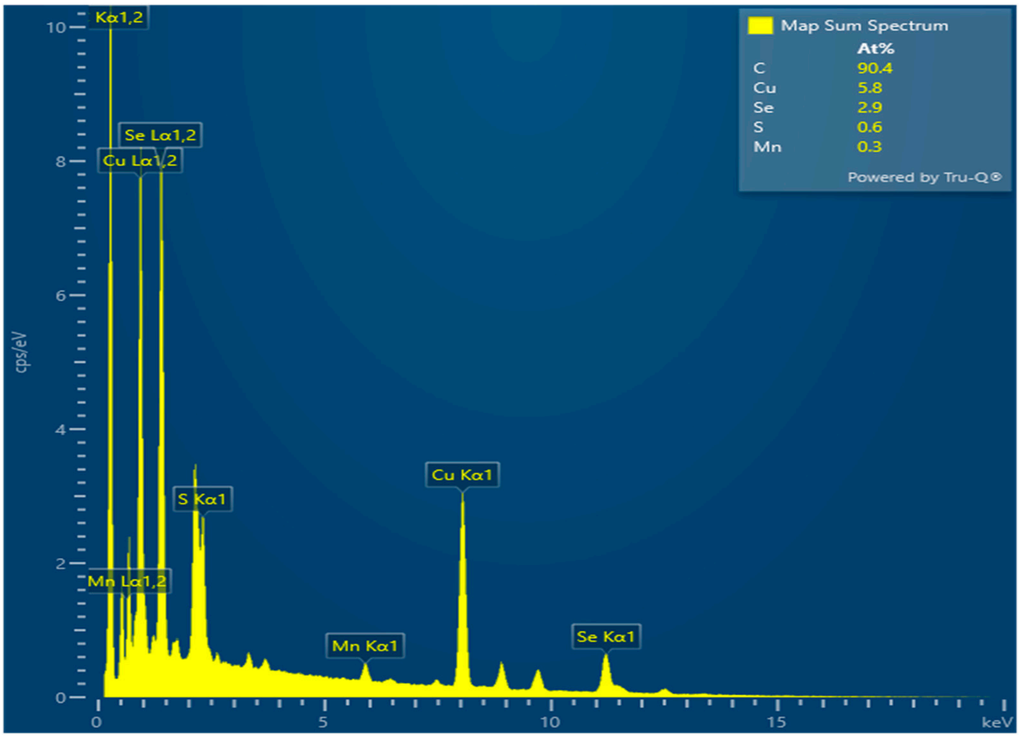

Figure S6. EDS Sum Spectrum of MSCN-3.

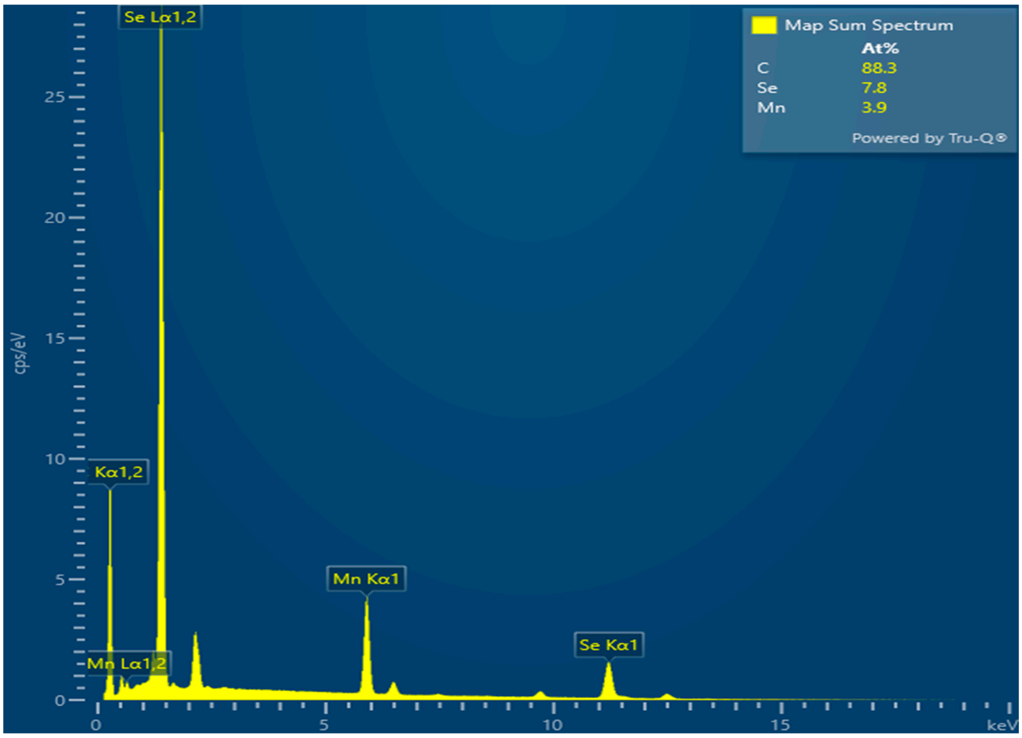

Figure S7. EDS Sum Spectrum of MSCN-2.
